# Effects of Using Aluminum Sulfate as an Accelerator and Acrylic Acid, Aluminum Fluoride, or Alkanolamine as a Regulator in Early Cement Setting

**DOI:** 10.3390/ma16041620

**Published:** 2023-02-15

**Authors:** Yihong Zhang, Yong Wu, Puyu Zhou, Zhiyuan Song, Yayun Jia, Weiyi Ouyang, Rafael Luque, Yang Sun

**Affiliations:** 1 Department of Applied Chemistry, School of Chemistry, Xi’an Jiaotong University, No. 28, Xianning West Road, Xi’an 710049, China; 2 Shanxi Jiawei New Material Co., Ltd., Taijia Village, Jiedian Town, Wanrong County, Yuncheng 044200, China; 3 Peoples Friendship University of Russia (RUDN University), 6 Miklukho Maklaya str., 117198 Moscow, Russia; 4 Universidad ECOTEC, Km 13.5 Samborondón, Samborondón EC092302, Ecuador

**Keywords:** aluminum sulfate, accelerator, setting time, mortar, strength

## Abstract

Aluminum sulfate was employed as the main accelerator in order to explore new non-chloride and alkali-free cement accelerators. Acrylic acid, aluminum fluoride, or alkanolamine were used as regulators to further accelerate cement setting. The setting time, compressive, and flexural strengths in cement early strength progress were detected, and both the cement (raw material) and hydrated mortar were fully characterized. The cement setting experiments revealed that only loading acrylic acid as the regulator would decrease the setting time of cement and increase the compressive and flexural strengths of mortar, but further introduction of aluminum fluoride or alkanolamine improved this process drastically. In the meantime, structural characterizations indicated that the raw material (cement) used in this work was composed of C_3_S (alite), while hydrated mortar consisted of quartz and C_3_A (tricalcium aluminate). During this transformation, the coordination polyhedron of Al^3+^ was changed from a tetrahedron to octahedron. This work puts forward a significant strategy for promoting the activity of aluminum sulfate in cement setting and would contribute to the future design of new non-chloride and alkali-free cement accelerators.

## 1. Introduction

Concrete appears to be a major demand for the construction industry in the 21st century due to its mechanical strength, structure adjustability as well as environmental compatibility [1,2]. Cement, only comprising 10–15% of mass of concrete, however, is a vital component of concrete because it transforms into a glue that can hold components of the mixture together and has sustained the building industry for a long time [1]. Taking into account the complicated composition of concrete as well as its comparatively simpler constitution of cement, improving the properties of cement seems to be a rational, available, and less costly approach to promote the quality of modern buildings [3].

Throughout history, ordinary Portland cement (OPC, denoted as standard cement in China) has played a dominant role in the construction industry for more than 170 years [1], with an annual output of 1500 million tons/year in 2000 [1]. Due to its great physical properties (e.g., compressive strength of 35 MPa [1]), ease of manipulation (modulus of elasticity 29 GPa [1]) as well as low cost, the application of OPC has become more and more popular worldwide. Meanwhile, the use of OPC as a model of building material has been widely adopted in architectural research [4,5].

Generally speaking, OPC mainly consists of sufficiently ground calcium silicate, which is obtained by mechanically mixing limestone (calcium source), sand (silica source) as well as small amounts of bauxite (aluminum source) and iron under heating at 1480 °C [1]. Simultaneously, the corresponding raw materials including limestone, marble, chalk, sand, or shale are abundantly supplied in the market. The final addition of gypsum yields OPC powders with a S/Ca mass ratio of about 5% [1].

**Scheme 1:**
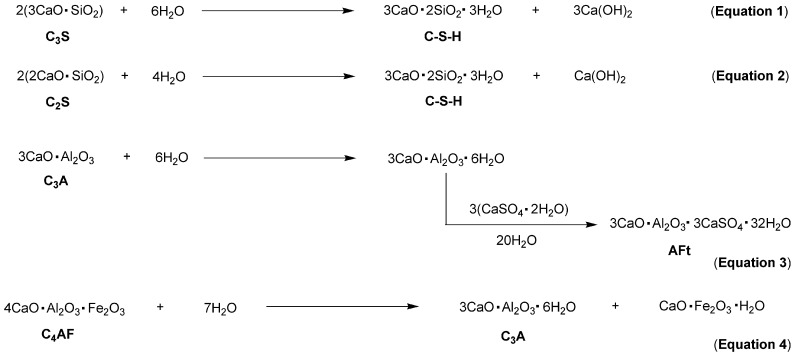
Hydration reactions of Portland cement.

On the basis of preparation, there are four components of OPC that affect the cement hydration process (C_3_S, C_2_S, C_3_A, and C_4_AF, Scheme 1) [4,6]. First of all, OPC is rich in phases such as C_3_S (alite, 3CaO⋅SiO_2_) and C_2_S (2CaO⋅SiO_2_), sharing 70–80% of the total mass, along with small amounts of C_3_A (tricalcium aluminate, 3CaO⋅Al_2_O_3_) and C_4_AF (tetracalcium aluminoferrite, 4CaO⋅Al_2_O_3_⋅Fe_2_O_3_) (Scheme 1) [4,6]. During hydration, not only C_3_S but also C_2_S reacted with H_2_O to yield a colloidal gel (C–S–H), which would link the cement particles and Ca(OH)_2_ (lime) together (Equations (1) and (2), Scheme 1) [6].

At the same time, the combination of C_3_A with CaSO_4_⋅2H_2_O (gypsum) in the presence of H_2_O produced AFt (calcium sulfoaluminate hydrate, 3CaO⋅Al_2_O_3_⋅3CaSO_4_⋅32H_2_O, Equation (3), Scheme 1), while the hydration of C_4_AF (ferrous-containing component) provided C_3_A and calcium-ferrous mixed oxide (Equation (4), Scheme 1) [4].

OPC still suffers from some shortcomings to date. The concrete derived from OPC shows poor to moderate durability, mainly due to its brittleness, low tensile strength, low ductility, and high permeability as well as weak resistance to acid [1]. Additionally, it has been reported that the content of C_3_S in OPC has been increased more than two times during the past years; the early strength of concrete was improved accordingly, but strength development after 28 days was decreased [7]. In particular, among the aforementioned reasons, sulfate (representing acid) attack appeared particularly deleterious to the concrete structure, usually leading to alkali–aggregate expansion reactions, freeze–thaw cycles, and corrosion [8].

The C–S–H network during OPC hydration is prone to degradation under sulfate attack [9]. In other words, the C–S–H network formed during OPC hydration is not thermodynamically stable. C–S–H shows an inclination to degrade to siliceous substances and calcium carbonate under some extreme climates such as acid rain or freezing [8]. On the other hand, a large amount of minerals and energy are consumed during the manufacture of OPC. Furthermore, a lot of carbon dioxide and other greenhouse gases are released. Meanwhile, the treatment of production waste and cost of transportation bring about heavy economic burdens [10].

Nowadays, the concept of green chemistry in cement design has attracted more and more interest. The key point lies in the trial of new and greener binding phases and composite materials, along with much few gas emissions as well as significantly decreased solid or liquid waste. For example, a large family of alkali-activated cements (pozzolanic cements) has aroused worldwide interest due to their content of aluminosilicate binding phase, where cement particles react after the addition of alkali in the early mixing stages [11]. Thus, the setting process of pozzolanic cements looked much faster than that observed in OPC [11]. Additionally, the use of pozzolanic cements avoids pollution derived from hydrogen fluoride (HF) or other volatile accelerators in cement setting [11].

Furthermore, the Al–Si–O inorganic polymers formed during setting showed a 3-dimensional network, which had slightly lower porosity, but yielded greater mortar strengths. Moreover, unlike using minerals or other freshly prepared materials in OPC production, the manufacture of pozzolanic cements employs a huge amount of industrial waste as feedstock including fly ash, slag, and alkaline sludge (red mud), greatly decreasing the environmental pressures [12].

Another new series of cements seem to be magnesia cements including a combination of magnesium oxide with magnesium chloride [13] as well as magnesia phosphate [14]. The former is emphasized by its good binding properties with high early strength, but suffers from poor weathering resistance [13]. However, high early strength, rapid setting, as well as satisfactory water and freeze–thaw resistance have been found in the application of the latter, but the main obstacle to a large-scale appears to be the high cost of phosphates [14]. Other new cements such as sulfoaluminate [15] and blended OPC-based cement [16] have also been put forward gradually, but there are no practical cements exceeding OPC to date after a comprehensive evaluation of the durability, chemical resistance, cost, energy-saving efficiency, and environmental friendliness.

In the construction of tunnels, bridges, or special buildings, the fast setting and hardening of concrete have a decisive influence on the accomplishment of engineering. Cement accelerators for both normal concrete and shotcrete (sprayed concrete) have emerged as the times require. From the viewpoint of composition and the corresponding effects, cement accelerators can be classified into chloride and non-chloride types [17]. In the early years, CaCl_2_, NaCl, and alkali chlorides were representatives of chloride-based accelerators, showing positive effects on setting and hardening of OPC, but currently, these chloride-based accelerators are not recommended due to the potential corrosion of Cl^-^ to a steel skeleton of concrete [18]. Therefore, non-chloride accelerators have emerged accordingly, involving nitrates, nitrites, thiocyanates, formates, and alkanolamines, which improve the hydration of cement without corrosion [19]. It was previously reported that Ca(NO_3_)_2_ could reduce the setting time under small dosage, and whose combination with triethanolamine or sodium thiocyanate could reach high early strength [20]. More than that, Ca(NO_2_)_2_ seems to be even more effective than Ca(NO_3_)_2_ on strength development, but its cost is much higher [21].

From another viewpoint, cement accelerators can be also divided into another two types including the alkali-containing and alkali-free ones. In engineering, alkali-containing accelerators usually show better compatibility between accelerators and cements than neutral or acidic accelerators, but cause 20–40% long-term strength loss, increased risk of alkali-induced hydrolysis, and subsequent precipitation of metal ions in shotcrete [22]. The alkali-free accelerators, however, usually have an alkali content lower than 1%, avoid alkali-induced hydrolysis in shotcrete, and decrease long-term strength loss [21].

**Scheme 2:**
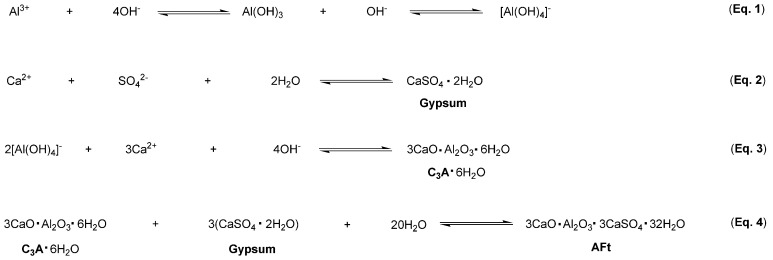
Aluminum sulfate-induced setting acceleration of Portland cement.

Aluminum sulfate (AS), usually in the form Al_2_(SO_4_)_3_⋅18H_2_O, is widely used as an accelerator due to its alkali-free and excellent acceleration nature, along with its acceptable cost [23]. The loading of AS promotes the hydration of C_3_A but retards that of C_3_S in OPC (Equations (1)–(4), Scheme 2), and both acceleration and retardation were improved when the loading of AS was increased [24]. The retardation of C_3_S hydration made by AS can be ascribed to the accelerated and large formation of AFt (ettringite, Scheme 1) on the surface of C_3_S (Equation (4), Scheme 2), which adsorb SO_4_^2-^, then hinder further hydration to C–S–H [24]. Meanwhile, due to the incorporation of SO_4_^2-^ derived from AS, the reaction shown in Equation (4) (Scheme 2) is prone to occur in the early stages, leading to high early strength, but long-term strength loss may occur [25].

AS can be dissolved in water as a single component yielding alkali-free liquid accelerators, but its high concentration solution seems unobtainable due to the low solubility of AS at room temperature (36.5 g AS/100 g H_2_O, 20 °C) [26], which finally leads to the precipitation or hydrolysis of Al^3+^, showing a poor acceleration effect [27]. In order to obtain a stable Al^3+^ solution with high concentration, many endeavors have been carried out, particularly regarding the combination of AS with a regulator.

Along this direction, the mixing of fluoride salts such as HF and NaF with AS produced fluoroaluminum complexes, which increased the solubility of Al^3+^, and F^-^ accelerates the setting of OPC [28]. A combination of AS with regulators such as gypsum, α-hemihydrate, β-hemihydrate, and anhydrite showed positive effects on the setting and hydration product morphology of OPC [24]. Furthermore, the introduction of inorganic and organic acids could inhibit the hydrolysis and aggregation of AS, and phosphoric acid looks more effective than lactic acid in stabilizing an AS solution [21]. Moreover, AS could be replaced with polyaluminum sulfate (Al(OH)_x_(SO_4_)_y_(H_2_O)_z_), probably due to its higher Al_2_O_3_ content and lower cost [21]. Additionally, amorphous Al(OH)_3_ seems to be another promising candidate because it could decrease the concentration of SO_4_^2-^, thus accelerating the hydration of C_3_A, however, Al(OH)_3_ is more expensive than AS [29].

It is also interesting to detect the effects of other organic regulators with coordinating atoms such as N and O. In practice, alkanolamines such as diethanolamine (DEA) and triethanolamine (TEA) are only ever used as a regulator, which not only promote the dissolution of AS, but also accelerates the hydration of OPC due to the coordination of N and O to Al^3+^ [30]. The application of some N- and O-containing polymers such as polyacrylamide also deserve attention because they can coordinate to Al^3+^, adjust the viscosity of concrete, and show much less environmental pressure.

In light of the low solubility of AS (36.5 g AS/100 g H_2_O, 20 °C), which would probably lead to the hydrolysis and precipitation of Al^3+^ [26], this work introduced acrylic acid (AA) as the main regulator in AS-facilitated cement setting. Herein, AA contained a carboxyl group, which could not only create an acid environment to inhibit the hydrolysis of Al^3+^, but also coordinate to Al^3+^, leading to a stable homogeneous solution. Next, aluminum fluoride (AlF_3_), DEA, and TEA were introduced respectively to further stabilize the Al^3+^ solution. During this process, both the setting time and mortar strength were tested, and both the cement (raw material) and mortars were comprehensively characterized to observe the hydration process. This work contributes to the design and application of new and effective non-chloride and alkali-free accelerators.

## 2. Experimental

### 2.1. Raw Materials

The aluminum sulfate (AS, Al_2_(SO_4_)_3_⋅18H_2_O, 99%) was purchased from Shanghai Aladdin Biochemical Technology Co. Ltd. Diethanolamine (DEA, 99%) and acrylic acid (AA, 99%) were both bought from Shanghai Macklin Biochemical Technology Co. Ltd. Triethanolamine (TEA, 99%) was commercially available from Shanghai Aladdin Biochemical Technology Co. Ltd. Aluminum fluoride (AlF_3_, 99%) was purchased from Alfa. Cement (PO 42.5) was bought from the China National Academy of Building Materials Science Co. Ltd. The Chinese ISO standard sand produced according to GB/T 17671 to measure the strength was purchased from Xiamen ISO Standard Sand Co. Ltd. (Xiamen, China).

### 2.2. Instruments and Methods

#### 2.2.1. Determination of Setting Time

The initial setting time (IST) and final setting time (FST) of cement paste were determined on a Vicat apparatus according to the Chinese standard JC 477-2005. First, AA (100 g) was combined with AS (350 g) in distilled water (150 mL). After vigorous stirring for 1 h at room temperature (20 °C), the accelerator was obtained (only 450 g of AS loaded for sample 1, Table 1), and its solid content was determined with a moisture meter (PC-16A).

In practice, cement (400 g) was combined with distilled H_2_O (140 g in total, involving water contained in accelerator determined by moisture meter as well as additionally added water) in a cement paste mixer (NJ-160A). The resulting mixture was stirred at low speed for 30 s. Then, the accelerator (32 g, dosage of 8% based on cement mass) was introduced through a weighing syringe (50 mL), and a regulator (AlF_3_, DEA or TEA, with pre-set loading amount, Table 1) was added subsequently. The resulting mixture was first stirred at low speed for 5 s, then at high speed for 15 s. Next, the cement paste obtained was immediately poured into a round (circular) mold and vibrated slightly. The surface of the cement paste was levelled through a scraper, leading to a smooth surface, then the IST and FST were determined every 10 s by using a Vicat apparatus.

The setting process was monitored by penetrating a needle of a fixed cross section into cement paste by using a constant force. IST was determined by counting the time between releasing the needle in a free fall manner and the needle reaching the preset depth (4 mm ± 1 mm above bottom). The FST was determined by counting the time between the end point of IST and the moment that needle could not penetrate any further.

#### 2.2.2. Determination of Compressive and Flexural Strengths

The compressive and flexural strengths of the cement mortar specimens were determined according to the Chinese standard JC 477-2005. In practice, distilled H_2_O (450 g in total, involving water contained in accelerator determined by moisture meter as well as additionally added water) and cement (900 g) were combined into a mixing bowl. The resulting mixture was immediately stirred at low speed for 30 s under a cement mortar mixer (JJ-5-). During the following low-speed stirring for 30 s, the Chinese ISO standard sand (1350 g) was gradually added. Then, the mixture obtained was stirred at high speed for 30 s, paused for 90 s, then further stirred at high speed for 30 s. After that, immediately, an accelerator (80 g, dosage of 8% based on cement mass) was introduced through a weighing syringe (100 mL), and regulator (AlF_3_, DEA or TEA, with pre-set loading amount, annotation a, Table 1) was also added. Then, the mixture obtained was further stirred at low speed for 5 s, then at high speed for 15 s. The cement mortar was transferred into a mold with the size of 40 mm × 40 mm × 160 mm (trial mold for cement mortar soft scouring) as soon as possible, then stored in a numerical control standard cement conservation box (HBY-40B-) with a temperature of 20 °C and a humidity of 90% for a pre-set incubation time (6 h and 24 h). Both the compressive and flexural strengths were tested on fully an automatic anti-folding and compression testing machine, whose max power was 300 kN, and the pressing speed was 48 N s^−1^.

#### 2.2.3. Characterizations

The chemical composition and valence state of the element on the sample surface (0–10 nm) was determined by X-ray photoelectron spectroscopy (XPS), using a Kratos Axis Ultra DLD with monochromatic Al Kα X-ray (1486.6 eV) as the lighting source, and the binding energy scale was calibrated by setting the C 1s peak at 284.8 eV. Furthermore, the peaks were fitted by using Gaussian–Lorentz (G/L) product function with 30% Lorentzian. The total elemental content and corresponding chemical composition of the cement were measured by X-ray fluorescence spectroscopy (XRF) using a Bruker S8 Tiger.

The phase of the sample was detected by wide-angle (2*θ* = 10–80°) X-ray diffraction (XRD) using Cu-Kα radiation (λ = 1.5418 Å), with an interval of 0.05° s^−1^. Thermogravimetric analysis (TGA) was carried out on an NETZSH TG 209C featuring a TASC 414/4 controller under nitrogen protection, with a heating rate of 10 °C min^−1^ at 30–600 °C.

## 3. Results and Discussion

### 3.1. Analysis of Setting Time and Mechanical Strength

**Figure 1:**
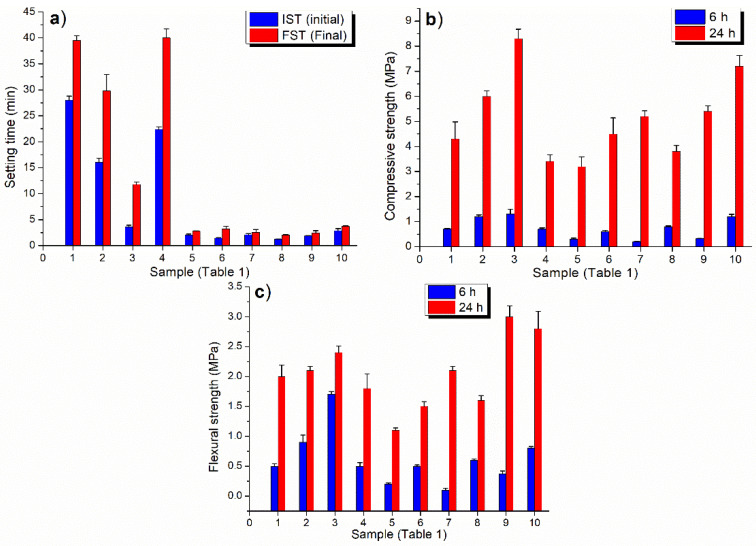
Histograms for the setting time of cement (**a**) and the mechanical strength of mortar (**b**,**c**) with SD bars under a different regulator.

Table 1 and Figure 1 show the setting time of the cement and early strength development of mortar caused by AS as well as the varied regulator. First of all, the introduction of AA as a regulator shortened both the IST and FST (samples 2 vs. 1, Table 1), and simultaneously improved the compressive and flexural strengths (samples 2 vs. 1, Table 1). This result indicated that the loading of AA may promote the dissolution of AS and inhibit the hydrolysis of AS in solution. In practice, it was found that AS could hardly be dissolved in water completely for the preparation of sample 1, leading to a two-phase mixture, while the addition of AA facilitated the dissolution of AS to a large extent. This phenomenon further emphasizes that solvated Al^3+^ of high concentration plays a key role in the acceleration of cement setting.

Next, in order to further accelerate cement setting, AlF_3_ was induced, and the corresponding outputs including IST, FST, compressive, and flexural strengths were improved (samples 3 vs. 2, Table 1). Herein, the combination of AS with AlF_3_ may give fluoroaluminum complexes, which improve the dissolution of Al^3+^ to accelerate cement setting [28]. However, due to the high cost of AlF_3_ and the potential environmental risks of F^-^ [28], the loading amount of AlF_3_ was halved, but both IST and FST were largely prolonged, and the compressive and flexural strengths decreased sharply (samples 4 vs. 3, Table 1). It seems that the favored fluoroaluminum complexes may appear when the concentration of F^-^ is high enough, otherwise, the coordination number is less, which may propel hydrolysis or the aggregation of Al^3+^ more than promote its dissolution.

Furthermore, it is of significance to detect the effects of DEA as a regulator, mainly due to its property of stabilizing Al^3+^, less environmental burden, non-toxic nature as well as comparatively lower cost [30]. In general, regardless of how much DEA was loaded, both the IST and FST were confined within 4 min (samples 5–7, Table 1). The compressive and flexural strengths of 6 h were varied within a small range, but those of 24 h were gradually improved as the loading amount of DEA was increased (samples 5–7, Table 1). It seems that DEA is highly responsive to cement setting, and even a small dosage would accelerate this process obviously, however, the compressive and flexural strengths after incubation of 24 h will increase along with the improvement in the DEA dosage. This tendency seems to be a new and complementary illustration of the effects of DEA compared to previous studies [30].

The application of TEA in cement setting may also arouse considerable interest due to its acceleration efficiency, commercial availability, non-toxic property as well as low cost [30]. In this work, unlike with DEA, TEA gradually showed prolonged IST and FST when its dosage was improved step by step (samples 8–10, Table 1). The compressive strength of mortar after being cured for 24 h was increased accordingly, while the compressive strength of 6 h and the flexural strengths were overall upward with small fluctuations (samples 8–10, Table 1). It was supposed that TEA coordinated to Al^3+^ affected the acceleration of cement setting, while the composition and structure of the resulting complex depended on the concentration of TEA, which facilitates both the setting time and mortar strength development. Obviously, the higher the TEA dosage, the better the strength development.

On the other hand, under a comprehensive evaluation of the setting time as well as the compressive and flexural strengths, a comparison of AlF_3_, DEA, and TEA can be summarized in the order of TEA > DEA > AlF_3_ for the present system. It would be of further interest to discuss the effects of DEA and TEA, because they showed structural systematic variability. In practice, the dosage of DEA showed little effect on the setting time, but the compressive and flexural strengths were increased when the dosage of DEA was improved (samples 5–7, Table 1), while the setting time, compressive and flexural strengths were all improved as dosage of TEA was promoted (samples 8–10, Table 1). This difference indicates that the complex of Al^3+^ with TEA seems to be more stable than that with DEA. Herein, TEA had an additional electron-donating hydroxyethyl compared to DEA, which made the electron cloud of nitrogen atoms on TEA denser, contributing to its stability. Nevertheless, although a coordinating difference between DEA and TEA existed, both DEA and TEA contributed greatly to the dissolution of AS, and the variation in the strengths after 24 h retained similar tendencies. Therefore, it can be seen that the main role of regulators such as DEA and TEA showing effects on setting more than hardening in the early cement setting process.

### 3.2. Analysis of Chemical Composition

**Figure 2:**
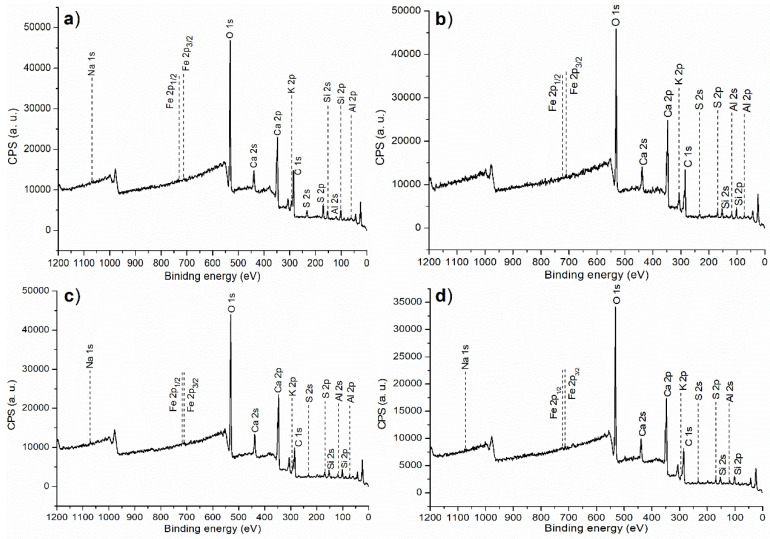
XPS survey scan: (**a**) Cement, (**b**) sample 2, (**c**) sample 7, and (**d**) sample 10.

With the performance characterizations obtained thus far, it would be of interest to carry out structural characterizations in order to further understand the influence of the accelerator and regulator in cement setting. The total chemical composition and ignition loss of cement are summarized in Table 2, indicating that the present raw material conformed to the characteristics of ordinary Portland cement [24]. Next, the elemental composition and chemical state of various samples were measured with X-ray photoelectron spectroscopy (XPS), providing a full observation on the sample surface. The binding energy and atomic composition of the sample are summarized in Table 3, while the XPS survey scan is shown in Figure 2.

Generally speaking, cement showed higher contents of S, Si, and K than all the mortars, but the variation tendencies of the other elements were not obvious (samples of cement vs. 2, 3, 7, 10, Table 3), indicating that the hydration of cement took place. The contents of Si and Al on sample 3 were higher than those on sample 2 (Table 3), revealing that AlF_3_ was incorporated into the cement during hydration (samples 3 vs. 2, Table 1), which further improved the occurrence of a C–S–H gel on sample 3 more than in sample 2 (Equations (1) and (2), Scheme 1). This result may also explain the differences in the setting time and mechanical strengths between samples 3 and 2 (Table 1).

Both samples 7 and 10 showed higher contents of Si and Al than sample 2 (samples 7, 10 vs. cement, Table 3), further emphasizing that DEA and TEA play a similar role as AlF_3_, where the Al^3+^ of AS was sufficiently stabilized, utilized, and incorporated into cement during hydration, obviously propelling the formation of C–S–H gel on the sample [30].

### 3.3. Analysis of Element Chemical State

**Figure 3:**
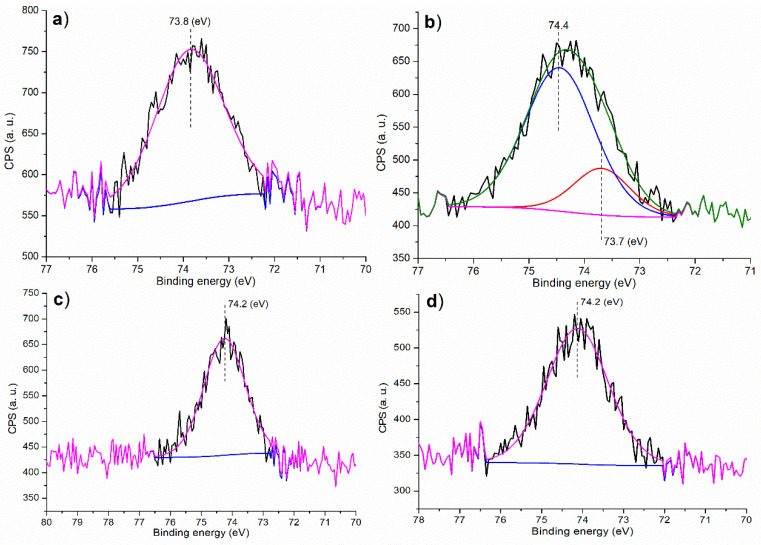
XPS measurements of the Al 2p region: (**a**) Cement, (**b**) sample 2, (**c**) sample 7, and (**d**) sample 10.

It is also interesting to discuss the chemical state of the element on the surface of the sample, which could contribute to understanding the cement hydration progress. The Al 2p regions of the samples of cement, 2, 7, 10 are provided in Figure 3. First of all, there was only one component that appeared at 73.8 eV in the Al 2p region of the sample cement, characterizing tetrahedral Al^3+^ of the Al_2_O_3_ phase [31], which was fixed in the C_3_A or C_4_AF phase of cement (Equations (3) and (4), Scheme 1).

Next, with the progress of hydration, the C_3_A component of cement was gradually transformed to AFt (Equation (3), Scheme 1), while C_4_AF was slowly changed to C_3_A (Equation (4), Scheme 1). During this process, it seems that tetrahedral Al^3+^ of the Al_2_O_3_ phase evolved into the octahedral Al^3+^ of Al_2_O_3_, which fixed in AFt, naturally leading to the shift of the Al 2p peak to a higher binding energy (Figure 3b–d vs. 3a).

**Figure 4:**
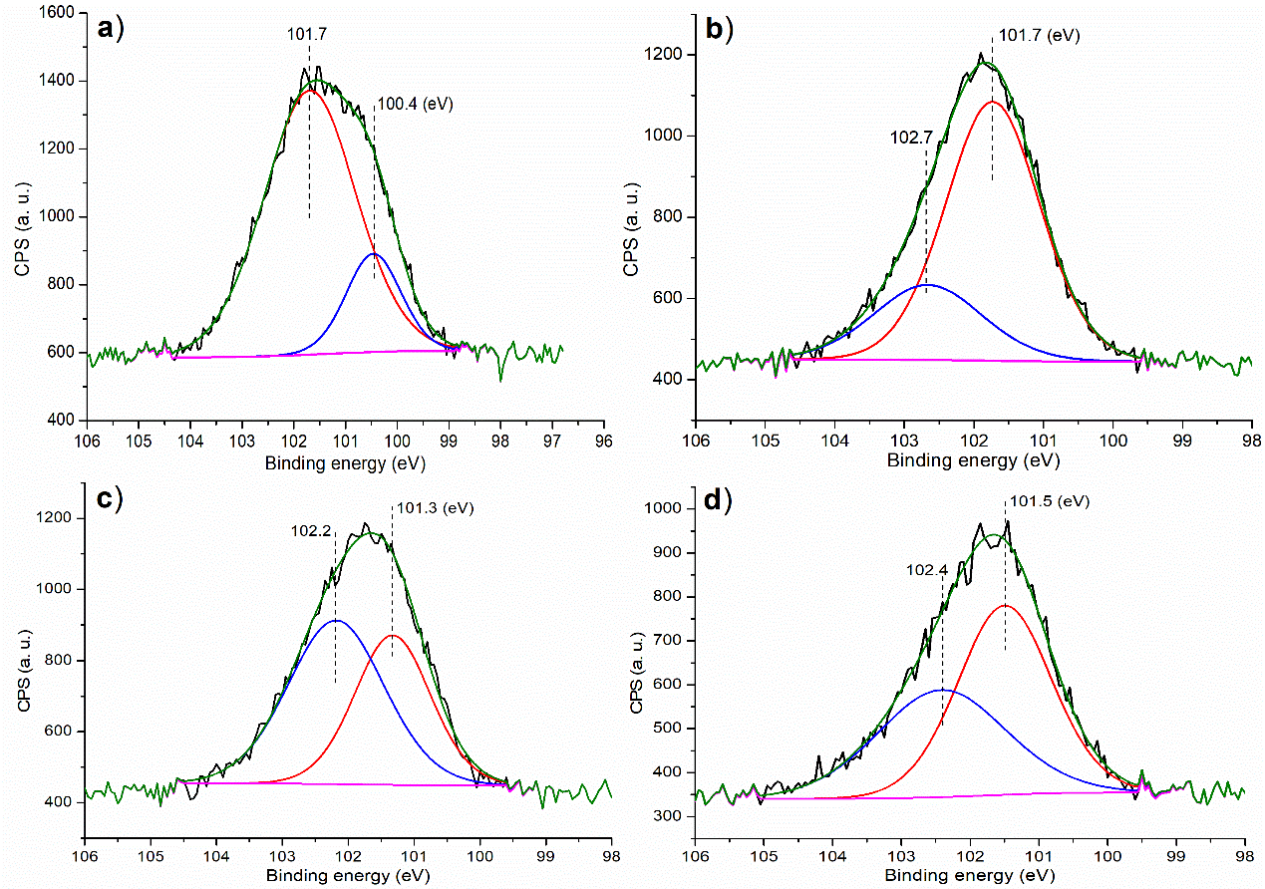
XPS measurements of the Si 2p region: (**a**) cement, (**b**) sample 2, (**c**) sample 7, and (**d**) sample 10.

Furthermore, cement showed two peaks at 101.7 and 100.4 eV (Figure 4a), corresponding to the Si 2p_1/2_ and 2p_3/2_ photoelectrons, respectively, indicative of the presence of the SiO_2_ phase in cement [32], probably referring to the SiO_2_ phase of C_3_S (Equation (1), Scheme 1). As cement hydration continued, peaks of both Si 2p_1/2_ and 2p_3/2_ shifted to higher binding energies (Figure 4b–d vs. 4a), characterizing the SiO_2_ component of the C–S–H colloidal gel.

**Figure 5:**
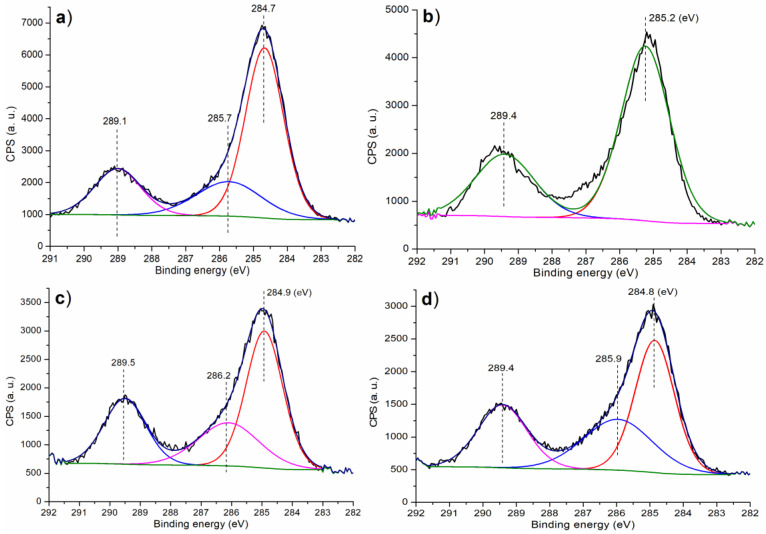
XPS measurements of the C 1s region: (**a**) Cement, (**b**) sample 2, (**c**) sample 7, and (**d**) sample 10.

Moreover, the detection of carbon-containing species may provide insights into the composition and properties of the ignition loss part of the cement or mortar. Three components occurred at 284.7, 285.7, and 289.1 eV in the C 1s region of cement (Figure 5a), representing carbons of saturated hydrocarbon (sp^3^ hybridization), the C–O bond as well as the carboxyl group, respectively [33]. In the meantime, cement showed no C 1s responses at binding energies higher than 292.0 eV, indicating that no polymeric carbon species such as polyethylene existed in cement [33]. Thus, it could be seen that the cement contained small organic molecules, which may come from minerals or mining processes.

When cement was combined with AS in the presence of AA and hydration was ignited (sample 2, Table 1), the resulting mortar showed two peaks centered at 285.2 and 289.4 eV (Figure 5b), suggesting carbons with the C–O bond and carboxyl group, respectively [33]. This phenomenon indicates that saturated hydrocarbon species (sp^3^ hybridization) of cement were almost oxidized into C–O or carboxyl groups during hydration. However, when DEA or TEA was introduced as a regulator to hydration, the response of saturated hydrocarbon appeared again (284.9 eV, Figure 5c; 284.8 eV, Figure 5d), being mainly ascribed to the residues of DEA or TEA during hydration (samples 7 and 10, Table 1).

**Figure 6:**
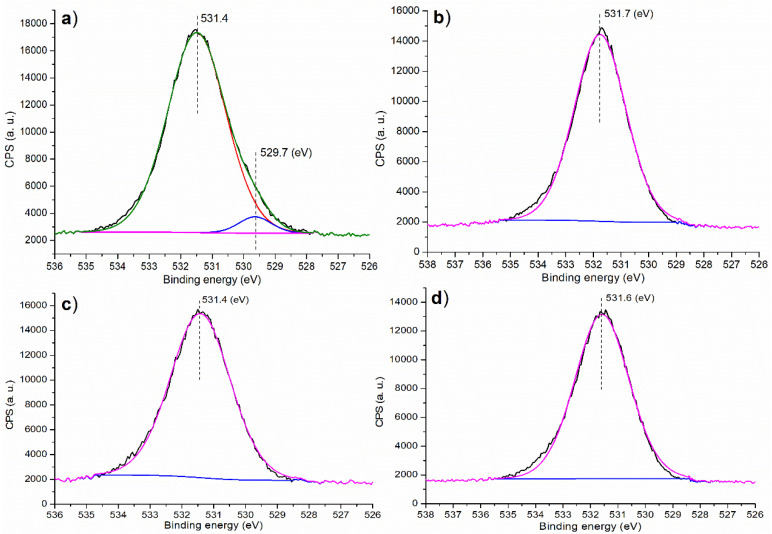
XPS measurement of the O 1s region: (**a**) Cement, (**b**) sample 2, (**c**) sample 7, and (**d**) sample 10.

Finally, the analysis of the O 1s region may provide structural information from the perspective of anions. Two peaks appeared at 529.7 and 531.4 eV in the O 1s region of cement (Figure 6a), probably corresponding to the oxygens of the CaO and SiO_2_ components of cement, respectively [34]. When cement was combined with AA and the regulator and hydration proceeded, the resulting mortars showed one peak at 531.4–531.7 eV (Figure 6b–d), characterizing the oxygen fixed in the C–S–H colloidal gel or C_3_A (Scheme 1).

### 3.4. Analysis of Crystallinity and Thermal Stability

**Figure 7:**
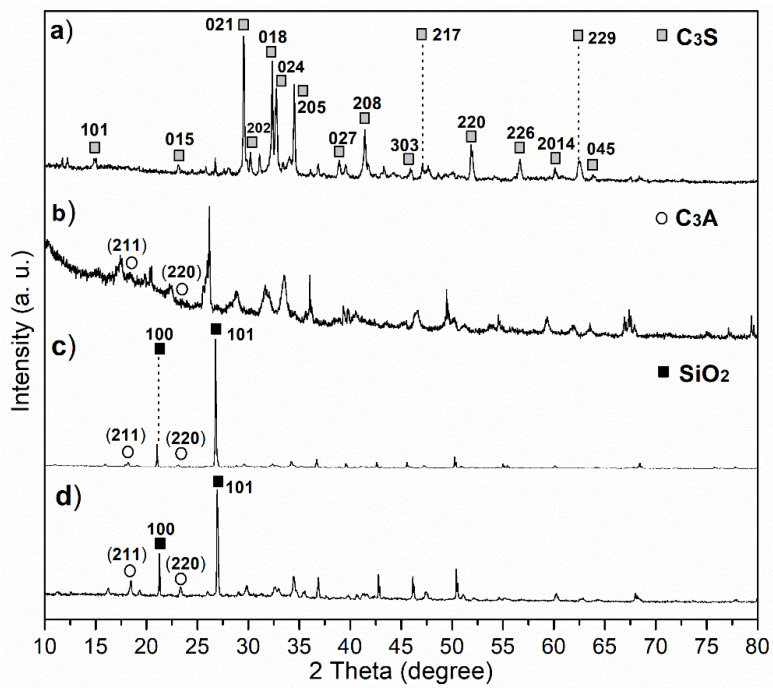
Wide-angle XRD patterns of the powdered samples: (**a**) Cement, (**b**) sample 2, (**c**) sample 7, and (**d**) sample 10.

XRD studies provide key information on the crystallinity and phase composition of the sample, which may illustrate or prove the results coming from XPS. First of all, cement only showed the diffractions of C_3_S (tricalcium silicate oxide, Ca_3_(SiO_4_)O, PDF No. 73-0599; grey cubes, Figure 7a), proving that the cement used as raw material in this work belonged to OPC. Herein, no other phases such as C_3_A or C_4_AF were detected by XRD (Figure 7a; Equations (3) and (4), Scheme 1), which could be ascribed to poor crystallinity or the low content of C_3_A or C_4_AF in cement.

As hydration proceeded in the presence of AS and AA (sample 2, Table 1), diffraction peaks of C_3_A were detected on the XRD of sample 2 (mayenite, Ca_12_Al_14_O_33_, PDF No. 09-0413; circles, Figure 7b), along with a set of broad diffractions, which probably means the intermediate of the C–S–H colloidal gel (Equation (1), Scheme 1). However, when hydration was accelerated by the AS, AA, DEA, or TEA (samples 7, 10, Table 1), the resulting mortars showed two series of diffraction peaks, the former belonging to C_3_A (mayenite, Ca_12_Al_14_O_33_, PDF No. 09-0413; circles, Figure 7c,d), while the latter was ascribed to SiO_2_ (quartz, PDF No. 09-0413; dark cubes, Figure 7c,d), coming from the Chinese ISO standard sand added in the measurement of mechanical strength (Section 2.2.2). On the other hand, it can be seen that the C–S–H colloidal gel was lacking the very characteristic diffraction peaks due to its low crystallinity.

**Figure 8:**
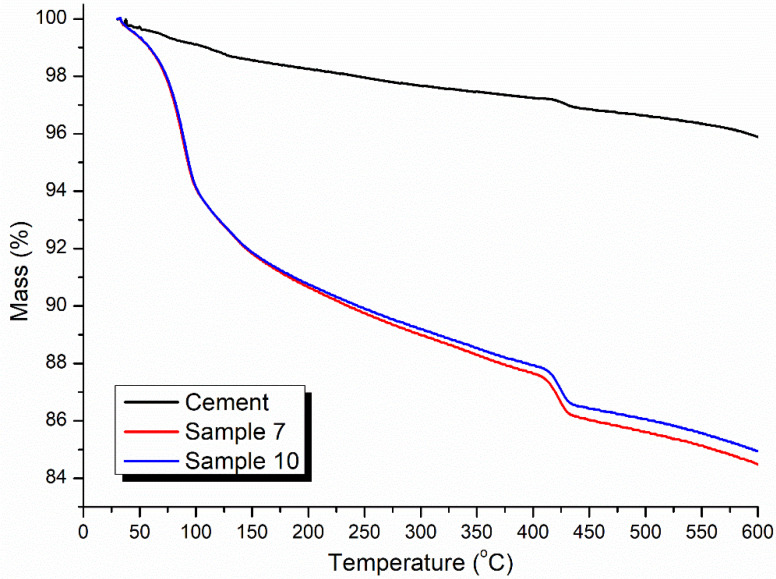
The TGA curves of the cement and mortar samples.

In addition, sample 10 showed higher compressive strength (24 h) and flexural strength (24 h) than both samples 2 and 7 (Table 1), while the surface contents of Al and O in sample 10 were higher than those found on samples 2 and 7 (Table 3). Furthermore, sample 2 showed a much poorer crystallinity of C_3_A compared to samples 7 and 10 (Figure 7b vs. Figure 7c,d), and sample 10 contained more C_3_A than sample 7 (H_211_(C_3_A)/H_100_(SiO_2_) = 1/3 for sample 10, Figure 7d; H_211_(C_3_A)/H_100_(SiO_2_) = 1/9 for sample 7, Figure 7c; H means the peak height). Therefore, it can be seen that the formation of C_3_A actually played a key role in the strength development of mortar. It was also reported that AS expedited the hydration process of C_3_A and produced ettringite (AFt, Equation (4), Scheme 2) [24]. Obviously, both DEA and TEA may facilitate this process, but TEA seemed better than DEA. The thermogravimetric analysis (TGA) of cement and hydrated mortars may provide information on the thermal stability of various components. At first, the weight loss of cement that appeared at 30–250 °C was 2.05% (black line, Figure 8), being ascribed to the ignition loss of cement. Cement further showed a weight loss of 1.32% at 250–500 °C (black line, Figure 8), which probably indicated the transformation of C_3_S on cement into isolated calcium oxide or aluminum oxide [35]. The last weight loss that appeared at 500–600 °C was 0.75% (black line, Figure 8), probably referring to the crystalline transformation of gypsum under heating [35].

Next, there were also three weight losses on the TGA curve of sample 7 (red line, Figure 7). The first one that appeared at 30–250 °C was 10.25%, covering the evaporation of volatile organic species (DEA) and dehydration [35]. The subsequent weight loss of 4.14% occurred at 250–500 °C, which could be attributed to the transformation of C_3_A into isolated metal oxides under heating [35], while the last one of 1.14% at 500–600 °C still seemed to be the crystalline transformation of gypsum [35]. Finally, sample 10 showed a coincident TGA curve to sample 7 at 30–150 °C (blue vs. red, Figure 7), suggesting that they contained the same amount of volatile components. In the range of 150–500 °C, sample 10 showed an appropriately parallel TGA line as sample 7 (blue vs. red, Figure 7), proposing the release of some involatile components or the crystalline transformation of gypsum under heating [35].

## 4. Conclusions

In this work, aluminum sulfate was used as the main accelerator, and acrylic acid, aluminum fluoride, diethanolamine, or triethanolamine were used as the regulator for the hydration of ordinary Portland cement. First of all, the introduction of acrylic acid as a regulator will shorten both the initial (IST) and final setting times (FST), while improving the compressive and flexural strengths compared to the pure aluminum sulfate-facilitated sample. Next, on the basis of acrylic acid, the introduction of aluminum fluoride would further decrease the setting time as well as increase the various mechanical strengths, but this effect was only exhibited when the loading amount of aluminum fluoride reached a high level.

On the other hand, in addition to acrylic acid, further doping of diethanolamine will decrease the setting time and increase the various mechanical strengths, and the more diethanolamine that is loaded, the more obvious is the effect. The loading of triethanolamine as a regulator showed a similar tendency. Overall, the accelerating and hardening effects of the regulators showed the order of triethanolamine > diethanolamine > aluminum fluoride.

Further characterizations revealed that the cement (raw material) was mainly composed of C_3_S (tricalcium silicate oxide), while the hydrated cement mortar contained C_3_A (mayenite, Ca_12_Al_14_O_33_). During early strength development, C–S–H formed, while the coordination polyhedron of Al^3+^ changed from a tetrahedron to octahedron in C_3_A. Additionally, the quartz phase could be detected by XRD during this period. In summary, this work provides an effective strategy for promoting the dissolution of aluminum sulfate and avoiding the hydrolysis and precipitation of Al^3+^ in cement hydration, while the acceleration of the cement setting was realized by the introduction of a regulator. This work contributes to the future design of new non-chloride and alkali-free cement accelerators, which may show high value in architectural fields requiring the fast setting and hardening of concrete.

## Figures and Tables

**Table 1:** Setting time of the cement and mechanical strength of mortar under different regulator loadings ^a^.

Sample	Regulator (Mass /g) ^b^	Setting Time/min ^c^		Compressive Strength /MPa ^d^		Flexural Strength /MPa ^d^	
		Initial	Final	6 h	24 h	6 h	24 h
1	None	28.0 ± 0.80 ^e^	39.50 ± 0.94	0.7 ± 0.03	4.3 ± 0.68	0.5 ± 0.04	2.0 ± 0.19
2	AA (8 g)	16.05 ± 0.81	29.79 ± 3.14	1.2 ± 0.07	6.0 ± 0.22	0.9 ± 0.12	2.1 ± 0.07
3	AA (8 g), AlF_3_ (3.2 g)	3.60 ± 0.35	11.80 ± 0.41	1.3 ± 0.19	8.3 ± 0.38	1.7 ± 0.05	2.4 ± 0.11
4	AA (8 g), AlF_3_ (1.6 g)	22.38 ± 0.50	40.0 ± 1.75	0.7 ± 0.05	3.4 ± 0.27	0.5 ± 0.06	1.8 ± 0.24
5	AA (8 g), DEA (1.6 g)	2.03 ± 0.20	2.80 ± 0.02	0.3 ± 0.04	3.2 ± 0.38	0.2 ± 0.02	1.1 ± 0.04
6	AA (8 g), DEA (3.2 g)	1.45 ± 0.06	3.21 ± 0.53	0.6 ± 0.05	4.5 ± 0.64	0.5 ± 0.02	1.5 ± 0.08
7	AA (8 g), DEA (6.4 g)	2.06 ± 0.23	2.53 ± 0.57	0.2 ± 0.01	5.2 ± 0.22	0.1 ± 0.03	2.1 ± 0.07
8	AA (8 g), TEA (1.6 g)	1.26 ± 0.04	2.03 ± 0.10	0.8 ± 0.03	3.8 ± 0.24	0.6 ± 0.02	1.6 ± 0.08
9	AA (8 g), TEA (3.2 g)	1.89 ± 0.04	2.46 ± 0.41	0.32 ± 0.01	5.4 ± 0.22	0.37 ± 0.05	3.0 ± 0.18
10	AA (8 g), TEA (6.4 g)	2.88 ± 0.41	3.71 ± 0.14	1.2 ± 0.09	7.2 ± 0.43	0.8 ± 0.03	2.8 ± 0.29

^a^ Experimental details as shown in Section 2.2.1.–2.2.2. ^b^ Data show the dosage for the initial and final setting times, which multiplied by 2.5 times yielded the data dosage to test the compressive and flexural strengths (Section 2.2.1.–2.2.2.). ^c^ As in Section 2.2.1. ^d^ As in Section 2.2.2. ^e^ Data format: average value ± SD (standard deviation), obtained from *t*-test.

**Table 2:** The total chemical composition of cement ^a^.

Composition	CaO	SiO_2_	Al_2_O_3_	Fe_2_O_3_	SO_3_	MgO	K_2_O	Na_2_O	Ignition Loss ^b^
Content (wt.%)	65.13	22.81	4.75	2.15	1.93	1.86	0.36	0.17	3.35

^a^ The total chemical composition of cement (PO 42.5) was determined by XRF. ^b^ Ignition loss was detected according to GB/T 34231-2017.

**Table 3:** Binding energies and atomic composition of the element on the surfaces (depth 1–10 nm).

Sample	C (1s)	O (1s)	S (2p)	Si (2p)	K (2p)	Ca (2p)	Al (2p)
Cement	285.50 (35.33) ^a^	531.80 (32.54)	169.80 (4.42)	101.80 (6.78)	293.0 (8.90)	347.80 (10.57)	73.80 (1.45)
2	284.80 (32.83)	531.80 (38.09)	168.80 (1.71)	101.80 (4.27)	293.0 (8.27)	346.80 (13.53)	73.80 (1.30)
3	284.80 (37.29)	531.80 (35.07)	168.80 (1.39)	101.80 (4.88)	293.0 (7.24)	346.80 (11.98)	73.80 (2.15)
7	284.80 (29.71)	530.80 (38.53)	168.80 (2.28)	101.80 (6.65)	293.0 (7.48)	346.80 (12.98)	73.80 (2.45)
10	284.80 (33.00)	531.80 (38.71)	168.80 (1.39)	101.80 (5.76)	293.0 (5.62)	346.80 (12.83)	73.80 (2.69)

^a^ Binding energy (eV), along with the atomic percentage (at%) in parentheses.

## Data Availability

We all authors would like to share our research data.
